# Repellent effects of Chinese cinnamon oil on nymphal ticks of *Haemaphysalis longicornis*, *Rhipicephalus haemaphysaloides*, and *Hyalomma asiaticum*

**DOI:** 10.1007/s10493-023-00855-7

**Published:** 2023-10-23

**Authors:** Yongzhi Zhou, Jie Cao, Yanan Wang, Badgar Battsetseg, Banzragch Battur, Houshuang Zhang, Jinlin Zhou

**Affiliations:** 1 Shanghai Veterinary Research Institute, Chinese Academy of Agricultural Sciences, Shanghai, 200241 China; 2 Institute of Veterinary Medicine, Mongolian University of Life Sciences, Zaisan, Ulaanbaatar, 17024 Mongolia

**Keywords:** Chinese cinnamon oil, Repellent, Tick, Y-tube bioassay

## Abstract

The repellent activity of Chinese cinnamon oil (*Cinnamomum cassia*) on nymphal ticks (*Haemaphysalis longicornis* Neumann, *Rhipicephalus haemaphysaloides* Supino, and *Hyalomma asiaticum* Schulze and Schlottke) was evaluated in a sample Y-tube bioassay. The results were based on the vertical migration of ticks during the host-seek phase and showed a dose-dependent repellent effect of Chinese cinnamon oil on the tested nymphs after 6 h. For *H. longicornis*, *R. haemaphysaloides*, and *H. asiaticum* at the concentrations (vol/vol) of 3, 3, and 1.5%, the repellent percentages over time were 68–97, 69–94, and 69–93%, respectively, which indicated strong repellent activities against ticks, similar to the positive control DEET (N,N-diethyl-3-methylbenzamide). Chinese cinnamon oil exerted the strongest effect on *H. asiaticum* nymphs. To our knowledge, this is the first study to investigate the repellent effects of Chinese cinnamon oil on ticks. Chinese cinnamon oil has considerable potential and should be developed as a practical tick repellent.

## Introduction

Ticks are the leading arthropod vector of viruses, bacteria, rickettsiae, protozoa, and fungi, causing diseases in non-human vertebrates and are ranked second to mosquitoes as a vector of human pathogens (Bior et al. 2002). In China, 117 species of ticks in seven genera have been recorded and > 60 tick species exhibit vector potential (Yu et al. 2015). The hard tick *Haemaphysalis longicornis* Neumann is native to central and eastern Asia, and has the capacity to become invasive when introduced to new areas (Hoogstraal et al. 1968). Recently, this species has expanded to 11 states in the USA since its first detection in 2017 (Piedmonte et al. 2021). *Haemaphysalis longicornis* transmits a wide range of pathogens, including *Anaplasma bovis*, *Babesia ovate*, *Babesia microti*, *Borrelia burgdorferi*, *Rickettsia japonica*, and *Theileria orientalis*, and it causes severe fever associated with the thrombocytopenia syndrome virus (SFTSV) (Wu et al. 2017; Piedmonte et al. 2021). *Rhipicephalus haemaphysaloides* Supino is a widespread tick species found in China and other south Asian countries. It transmits animal babesiosis and human Kyasanur Forest disease (Zhou et al. 2006). *Hyalomma asiaticum* Schulze and Schlottke is one of three hard tick species widely distributed throughout northwest China and central Asia. It is a vector of *Babesia* spp. and *Theileria* spp. in animals and humans (Wang et al. 2016).

A wide variety of chemicals and drugs have been used to control tick infestations; however, acaricide applications have limited efficacy in reducing tick infestations and are often accompanied by serious drawbacks, including the selection of acaricide-resistant ticks, environmental pollution, and the contamination of milk and meat products with drug residues (Graf et al. 2004). Studies on the repellent action and acaricidal activity of products with plant origins (e.g., essential oils and plant extracts) have been increased and aim to replace or combine the use of synthetic chemical products (Benelli and Pavela 2018). These plant products often contain a variety of compounds with different modes of action, which hinder the selective development of resistant strains, as well as have a low environmental impact due to their biodegradability (Zeringóta et al. 2013). Botanical essential oils are complex mixtures of 20–60 low-molecular weight metabolites produced by aromatic plants, typically characterized by two or three major terpene or terpenoid components (Bakkali et al. 2008). Previous studies highlighted the tick-repellent efficacy of different essential oils and found that they hold considerable potential for development as practical tick repellents (Meng et al. 2016; Štefanidesová et al. 2017; Soutar et al. 2019). Cinnamon oil has been used in popular medicine for many years due to its fungicidal, bactericidal, and insecticidal properties (Ooi et al. 2006; Bassolé and Juliani 2012; dos Santos et al. 2017). Cinnamon oil is derived from two main species of *Cinnamomum*, *C. verum* (syn. *C. zeylanicum*) and *C. cassia* (syn. *C. aromaticum*). The cinnamon oil of *C. verum* is popular throughout the world, whereas the cinnamon oil from *C. cassia* (Chinese cinnamon oil) has been traditionally used in Chinese medicine. Currently, investigations on the repellency of cinnamon oil remain scarce, and only the limited effectiveness of *C. verum* oil has been observed (Meng et al. 2016; Benelli and Pavela 2018).

These two *Cinnamomum* species vary geographically, and biochemically. *Cinnamomum verum* is native to India and Sri Lanka, whereas *C. cassia* is native to China, Indonesia, Laos, and Vietnam. The major volatile compounds present in the bark of *C. verum* include cinnamaldehyde (65–80%), eugenol, and trans-cinnamic acid (5–10%) (Bisset and Wichtl 2001). However, there is little to no eugenol present in *C. cassia* (Ooi et al. 2016). Additionally, coumarin is present in *C. cassia* in very small amounts (0.45%), whereas it is completely absent in *C. verum* (Archer 1988).

In this study, the application of Chinese cinnamon oil, its potential importance in pharmacology and economics, and its repellent effects on three dominant species of nymphal ticks in China were investigated. Our findings will serve as a helpful resource for the development of Chinese cinnamon oil as a practical repellent against ticks.

## Materials and methods

### Ticks

Colonies of *R. haemaphysaloides*, *H. asiaticum*, and *H. longicornis* ticks were established at the Shanghai Veterinary Research Institute, Chinese Academy of Agricultural Sciences, Shanghai, China (Wu et al. 2017). Tick rearing was performed in the laboratory in the incubator at L12:D12 photoperiod, 25 °C and 92% relative humidity. Ticks openly fed on New Zealand white rabbits. All experimental tick nymphs were derived from post-blood meal larvae that fed on the rabbits and were 2–3 weeks old after molting from engorged larvae.

### Chinese cinnamon oil

Hydro-distilled *C. cassia* essential oil (batch number: 20,200,606) was purchased from the Ji’an Lvyuan Natural Perfume Oil Refinery (Jiangxi, China). The oil was tested at various concentrations diluted in 95% ethanol (solvent).

### N,N-diethyl-3-methylbenzamide (DEET)

DEET (C_12_H_17_NO, 97% pure; Sigma-Aldrich, St. Louis, MO, USA) was tested at various concentrations diluted in 95% ethanol.

### Gas chromatography-mass spectrometry (GC/MS) analysis

Chinese cinnamon oil was analyzed using an Agilent Technologies Gas Chromatograph (7890B; Agilent Technologies, Santa Clara, CA, USA) coupled to an Agilent Technologies Mass Selective Detector (5977 A). The column used for the analysis was a HP-5 capillary column (30.0 m × 250 μm i.d. × 0.25 μm) using helium as the carrier gas. The injector temperature was 250 °C and the split flow was 1.5 mL/min. The GC oven temperature was set to 60 °C for 4 min and programmed to increase to 280 °C at a rate of 4 °C/min, then maintained at 280 °C for 64 min. The MS was taken at 70 eV with a mass range of 35–425. Oil components were identified by comparing the mass spectrum of each peak with a mass spectra library (Wiley Registry of Mass Spectral Data, 7th edn.; John Wiley & Sons, New York, NY, USA). The relative percentages were calculated from the total ions chromatograms (TIC).

### Tick repellency bioassay using a sample Y-tube

**Fig. 1:**
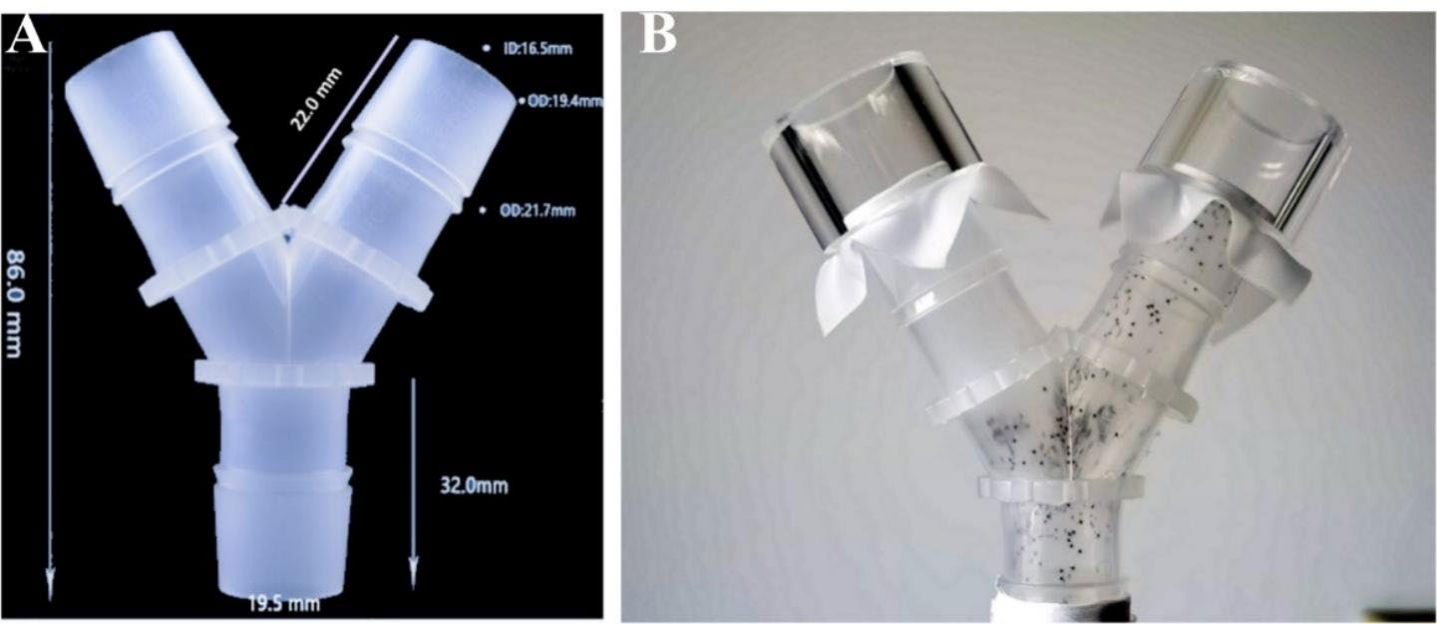
(**A**) The Y-tube apparatus. Whole body length: 86 mm; length of stem: 32 mm; length of arm connected to an odour source (= the arm where ticks were counted): 22 mm. ID, internal diameter; OD, outer diameter. (**B**) Example of an experiment with the vertical Y-tube: the odour source in the left arm was a repellent, the right arm contained the negative control

All experiments were conducted in ventilated room at room temperature. Only ticks responding to human breath by vigorously walking prior to the experiment were used. Based on the ambush behavior of most nymph ticks with a typical vertical movement (Adenubi et al. 2018), all tests were conducted using a still-air vertical Y-tube apparatus, modified from a standard Y-tube olfactometer (Zeringóta et al. 2020) (Fig. 1a). The whole Y-tube apparatus was 86 mm long, and the length of the arms where ticks were counted was 22 mm (Fig. 1a).

About 80–100 tick nymphs were placed in a tube, which was connected to the stem opening of the Y-tube. To evaluate the vertical movement of ticks, ticks were given a choice between a test sample arm and solvent blank arm (95% ethanol). The 15 uL test sample or solvent was added on Whatman Grade 1 filter paper discs (1 cm diameter), and then placed on a nylon membrane covering the arm openings, and the test was to start at once (Fig. 1b). Tests were independently conducted at oil concentrations of 0.75, 1.5, 3, 6, and 12% (vol/vol); a solvent blank arm (95% ethanol) was used as negative control. The experiment was executed 3×. Fresh ticks, Y-tubes, and chemicals were used for each run. Observations were made after 20, 40, 60, 120, 180, 240, 300, and 360 min. The results are expressed as % tick repellence to the test samples using the equation: [(no. ticks in the solvent arm – no. ticks in the sample arm)/no. ticks in the solvent arm] × 100%.

### Data analysis

Data from repellent activity experiments were analyzed by a two-way ANOVA followed by Dunnett’s multiple comparisons test (α = 0.05). All statistical analyses and data graphing were conducted using Prism v.6.01 software (GraphPad Software, San Diego, CA, USA).

## Results

### Components of Chinese cinnamon oil

**Fig. 2:**
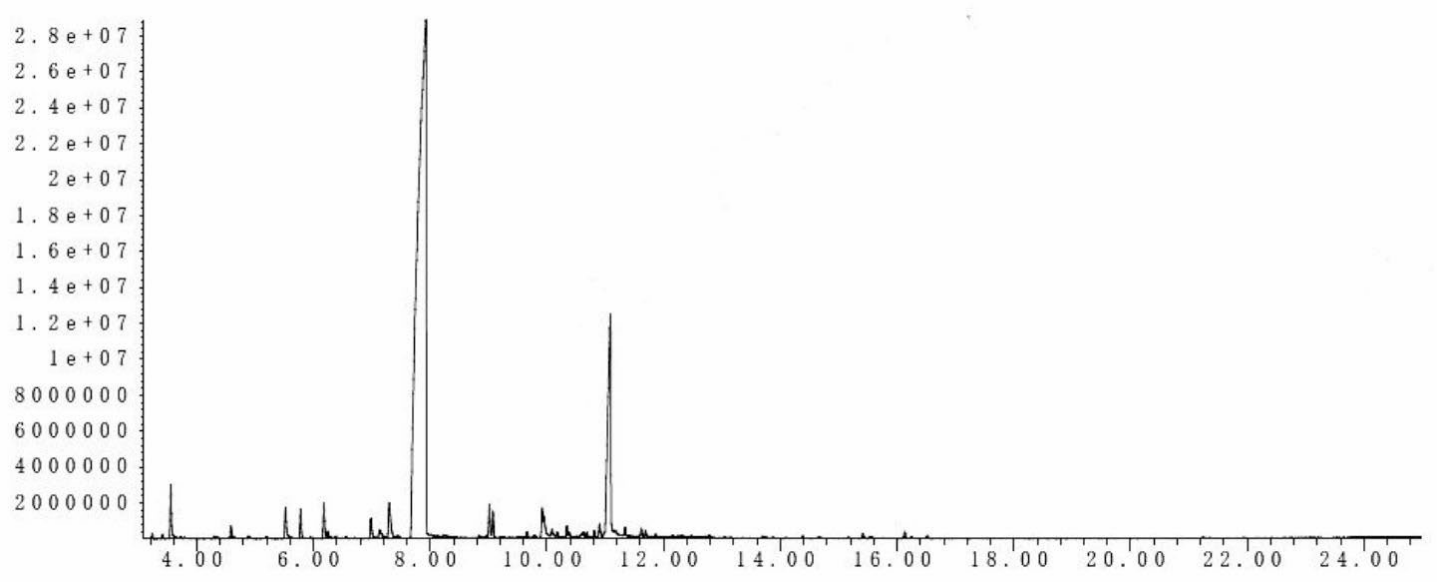
GC/MS chromatogram of *Cinnamomum cassia* essential oil. The major peak was trans-cinnamaldehyde (73.0%), followed by o-methoxy-cinnamaldehyde (11.7%)

GC/MS analysis identified a total of 52 components in the essential oil, which represented 99.999% of the total composition (Table 1). Trans-cinnamaldehyde (73.0%) was the major peak, followed by o-methoxy-cinnamaldehyde (11.7%) (Fig. 2). Small amounts of other constituents, including o-methoxy-benzaldehyde (1.4%), benzaldehyde (1.2%), and terpenoids were also identified (Table 1).

**Table 1:** Constituents identified in the oil of *Cinnamomum cassia* by GC-MS

Peak no.	Constituents	Area %	Peak no.	Constituents	Area %
1	α-pinene	0.095	27	trans-α-mandarin oil alkenes	0.101
2	camphene	0.075	28	cinnamyl acetate	0.686
3	benzaldehyde	1.244	29	coumarin	0.885
4	(-)-β-pinene	0.035	30	cis-o-methoxy cinnamaldehyde	0.37
5	4-isopropyltoluene	0.043	31	alloaromadendren	0.225
6	(+)-limonene	0.034	32	γ-chlamydia	0.287
7	salicylaldehyde	0.333	33	α-curcumin	0.154
8	acetophenone	0.086	34	(+)-ledene	0.139
9	guaiacol	0.052	35	α-chlamydia	0.144
10	phenethylol	0.902	36	β-bisabolene	0.147
11	o-methylacetophenone	0.597	37	γ-cadinene	0.177
12	phenylpropyl aldehyde	0.847	38	delta-cadinene	0.392
13	2-octanol	0.224	39	O-methoxy-cinnamaldehyde	11.666
14	α-terpineol	0.043	40	α-dihydro calamus alkenes	0.403
15	cis-cinnamaldehyde	0.612	41	trans-orange tertol	0.445
16	3-phenyl alcohol	0.464	42	spathulenol	0.307
17	O-methoxy-benzaldehyde	1.436	43	caryophyllene oxide	0.189
18	phenylethyl acetate	0.122	44	myristic aldehyde	0.104
19	trans-cinnamaldehyde	73.015	45	T-cadinol	0.139
20	2’-methoxyacetophenone	0.206	46	alpha-bisabolol	0.089
21	cinnamyl alcohol	0.257	47	benylate	0.124
22	eugenol	0.059	48	ketone	0.051
23	(+)-cyclic europene	0.063	49	phenethyl benzoate	0.069
24	O-methoxyphenyl ketone	0.762	50	(+)-rimuene	0.128
25	(-)-α-cubano	0.574	51	natural verticillol	0.16
26	β- bamboos	0.177	52	kaura-16-ene	0.061
				Total	99.999

### Repellent activity of Chinese cinnamon oil

**Fig. 3:**
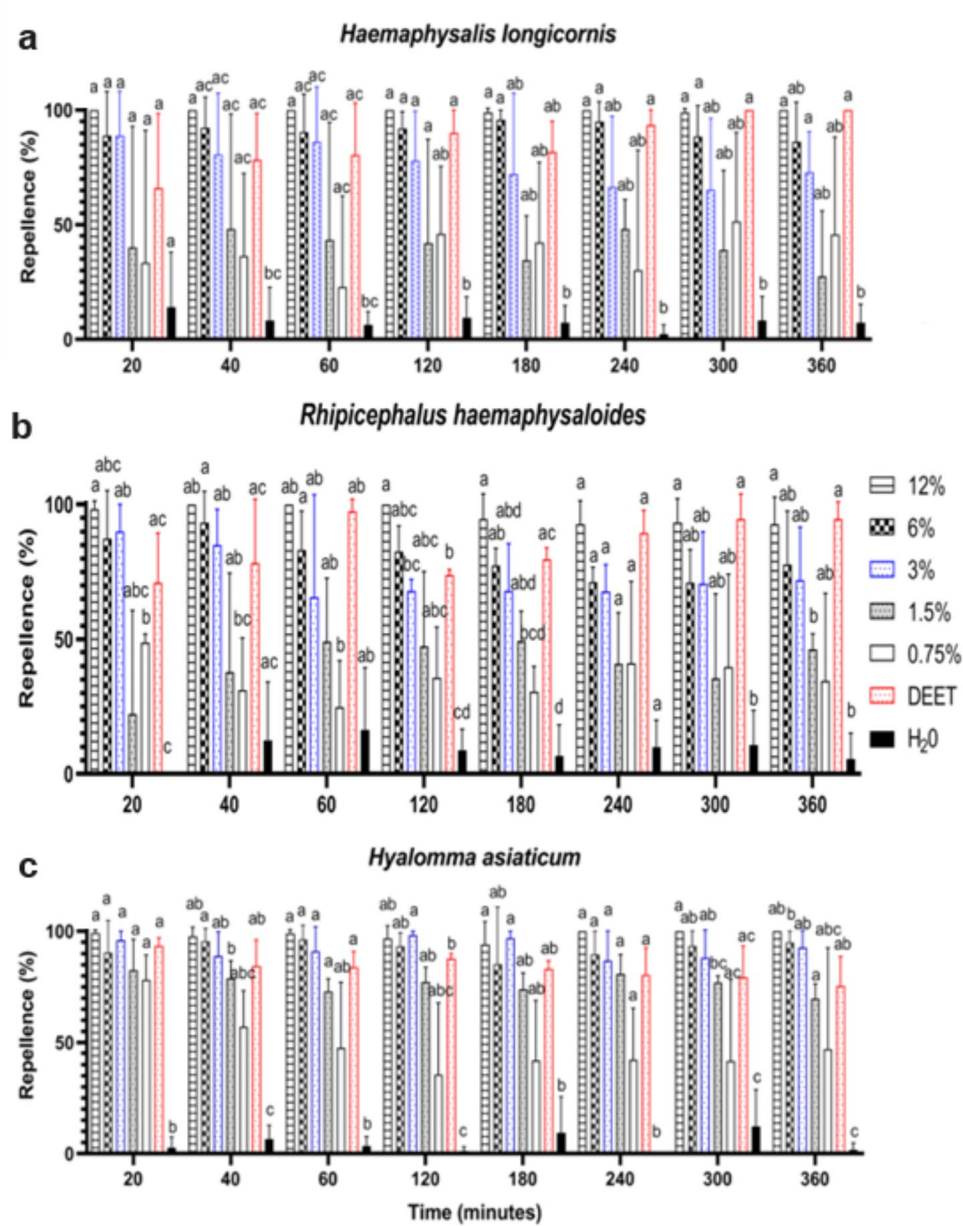
Dose response tests of Chinese cinnamon oil (0.75–12%) against (**a**) *Haemaphysalis longicornis*, (**b**) *Rhipicephalus haemaphysaloides*, and (**c**) *Hyalomma asiaticum*: mean (± SE) repellency (%) and longevity (20–360 min). Means within a time and within a tick species capped with different letters are significantly different (Dunnett’s multiple comparisons test: *P* < 0.05)

Preliminary assays using the sample Y-tube in a vertical position showed that the equipment was adequate to proceed with the experiments. Nymphs of all three tick species exhibited ambush behavior with a typical vertical movement to light. No significant changes to the control (water or 95% ethanol solvent) were detected (2-way ANOVA, *H. longicornis*: F_1.29,2.57_ = 0.095, P = 0.84; *R. haemaphysaloides*: F_1.77,3.54_ =0.21, P = 0.80; *H. asiaticum*: F_1.27,2.55_ = 1.231, P = 0.38). The standard repellent, DEET (20%), for tick species exhibited 65–100, 75–93, and 70–97% repellency over time for *H. longicornis*, *R. haemaphysaloides*, and *H. asiaticum*, respectively (Fig. 3).

For *H. longicornis*, at concentrations of 0.75 and 1.5% and at all evaluation times, no significant difference was detected when compared to the control, which indicated that there were no repellent activities against the tested ticks at these two concentrations. The repellence rates at 1.5 and 0.75% cinnamon oil were both < 50%. The mean repellence levels of the other test groups after 6 h significantly differed from the control (Fig. 3a). At 3, 6, and 12%, repellence rate was > 68, 87, and 97%, respectively, which indicated strong repellent activities against ticks, similar to the positive control (DEET, 65–100% repellence rate) (Fig. 3a).

For *R. haemaphysaloides*, at 0.75% after 1, 2, 5, and 6 h and at 1.5% after 5 and 6 h, no significant differences were detected when compared to the control; the repellence rates of 1.5 and 0.75% were both < 50%. The mean repellence levels of the other test groups after 6 h significantly differed from the control (Fig. 3b). At 3, 6, and 12%, the repellence rate was > 69, 73, and 94%, respectively, which indicated strong repellent activities against ticks, similar to the positive control (DEET, 70–94% repellence rate). A repellence rate of > 90% was only observed at the highest concentration (12%), which differed significantly from 1.5 to 0.75% in all cases (Fig. 3b).

For *H. asiaticum*, the repellence rate at 0.75% was < 50%, which was also significantly lower compared to the positive control (DEET), except after 6 h. The mean repellence levels after 6 h at 1.5% 3, 6, and 12% were > 69, 86, 85, and 93%, respectively, which indicated strong repellent activities against ticks, similar to the positive control (DEET, 75–93% repellence rate) (Fig. 3c).

Clearly, Chinese cinnamon oil had a dose-dependent repellent effect on nymphs of all three tick species (Fig. 3).

## Discussion

DEET is a gold standard repellent used against a large spectrum of arthropods, including several tick species (e.g., *I. ricinus*, *Ixodes scapularis*, *Amblyomma americanum*, *A. sculptum*, *R. sanguineus*, and *R. microplus*) (Carrol et al. 2014; Büchel et al. 2015; Lima et al. 2016; Ferreira et al. 2017). Owing to problems associated with the use of DEET and its potential risk to human health, safer and more effective alternatives are being investigated by the scientific community. Essential oils and other natural products are potential repellents that may be safer to apply, because they could have fewer side effects after regular use (Bissinger and Roe 2010).

To our knowledge, this is the first study to investigate the repellent effects of Chinese cinnamon oil on ticks. Our results showed that Chinese cinnamon oil had excellent repellent activities after 6 h of observation on *H. longicornis*, *R. haemaphysaloides*, and *H. asiaticum*. Chinese cinnamon oil exerted the strongest effects on *H. asiaticum* among the three tick species. Previously, *C. verum* cinnamon oil did not show good repellency (Meng et al. 2016). Besides a different bioassay with a different criterion for repellence, this difference from our findings may be due to major volatile compound differences between Chinese and *C. verum* cinnamon oil. In this study, the GC/MS analysis of Chinese cinnamon oil identified 52 components, including trans-cinnamaldehyde (73.0%), o-methoxy-cinnamaldehyde (11.7%), and small amounts of other constituents. Little eugenol was present in Chinese cinnamon oil, but is an important constituent in *C. verum* cinnamon oil (Bisset and Wichtl 2001).

Repellence is detected by significantly reduced number of ticks entering the treated zone compared to the negative control. Various methods are used to estimate repellence rates, including, amongst others, Petri dishes, tick climbing, and Y-tube olfactometers (Adenubi et al. 2018). Different types of olfactometers have been used to test candidate repellents against ticks. Olfactometer tests range from the rather simple Y-tube assay to highly sophisticated tracking systems using a locomotion compensator (Adenubi et al. 2018). A simple Y-tube apparatus modified from an olfactometer was used in our study for evaluating tick repellency. The Y-tube apparatus was deemed to be adequate for experimentation, as all nymphal ticks exhibited climbing behavior with a typical vertical movement and the number of ticks in both arms of the vertically positioned Y-tube was similar. No significant response to the solvent control and negative control (water) were detected in any test group, but the standard repellent (DEET) showed effective repellency. The Y-tube apparatus allows for about 80–100 ticks to be assayed, as well as rules out inactive ticks, which is useful for acquiring accurate data. Additionally, this method is convenient for operation and safe for tick escape.

Currently, essential oils represent an alternative tick-bite prevention method for people who may object to using synthetic repellents (Dietrich et al. 2006; Shapiro 2012). Many essential oils are known to be safe at low doses (diluted to 0.05–4%) (Price and Price 2007). *Cinnamomum cassia* has been reported to possess many pharmacological properties such as antibacterial, anticancer, antidiabetic, antifungal, neuroprotective and antileishmanial activity (Zhang et al. 2019; Afrin et al. 2019). In a recent study, the oil of *C. cassia* was investigated and exhibited good acaricidal activity against the larvae and nymphs of *H. longicornis*; however, no toxic effect has been found against terrestrial invertebrates (Nwanade et al. 2021). The climbing behavior of ticks in pastures is a strategy used for locating and attaching to hosts. Therefore, the deterrence of this climbing behavior by Chinese cinnamon oil is an indication of its repellent effect on ticks. Results from these experiments indicated a dose-dependent repellent effect of Chinese cinnamon oil after 6 h on *H. longicornis*, *R. haemaphysaloides*, and *H. asiaticum* in concentrations of > 3, 3, and 1.5%, which resulted in repellence rates of > 68, 69, and 69%, respectively. Therefore, Chinese cinnamon oil showed strong repellent activities against ticks, which was similar to the positive control (DEET). Clearly, Chinese cinnamon oil is able to repel ticks at low doses and would be preferable when considering regular or daily use.

It is clear that the repellent effect of Chinese cinnamon oil became weaker over time, as with other essential oils, and so does not have a long duration, which is a characteristic of ideal repellents. In this respect, it is important to focus on testing essential oils adapted through encapsulation, which may prolong the duration of their efficacy considerably (Banumathi et al. 2017). The nano-formulation of repellents through green synthesis routes can also represent a novel and appealing alternative (Benelli and Pavela 2018).

## Conclusion

The present study demonstrated that Chinese cinnamon oil exerted strong repellent effects against nymphs of *H. longicornis*, *R. haemaphysaloides*, and *H. asiaticum* when compared to the most commonly used repellent, DEET. Chinese cinnamon oil had a dose-dependent repellent effect on these ticks after 6 h. Chinese cinnamon oil has considerable potential as a practical tick repellent.
